# Involvement of the chloroplast gene ferredoxin 1 in multiple responses of *Nicotiana benthamiana* to *Potato virus X* infection

**DOI:** 10.1093/jxb/erz565

**Published:** 2019-12-24

**Authors:** Xue Yang, Yuwen Lu, Fang Wang, Ying Chen, Yanzhen Tian, Liangliang Jiang, Jiejun Peng, Hongying Zheng, Lin Lin, Chengqi Yan, Michael Taliansky, Stuart MacFarlane, Yuanhua Wu, Jianping Chen, Fei Yan

**Affiliations:** 1 State Key Laboratory for Managing Biotic and Chemical Threats to the Quality and Safety of Agro-products, Institute of Plant Virology, Ningbo University, Ningbo, China; 2 Key Laboratory of Biotechnology in Plant Protection of MOA and Zhejiang Province, Institute of Virology and Biotechnology, Zhejiang Academy of Agricultural Sciences, Hangzhou, China; 3 Department of Plant Protection, Shenyang Agriculture University, Shenyang, China; 4 Ningbo Academy of Agricultural Sciences, Ningbo, China; 5 College of Plant Protection, Fujian Agriculture and Forestry University, Fuzhou, China; 6 The James Hutton Institute, Cell and Molecular Sciences Group, Invergowrie, Dundee, UK; 7 Shemyakin-Ovchinnikov Institute of Bioorganic Chemistry of the RAS, Moscow, Russia; 8 Australian National University, Australia

**Keywords:** ABA, ferredoxin 1, p25, PD callose, *Potato virus X*, SA

## Abstract

The chloroplast protein ferredoxin 1 (FD1), with roles in the chloroplast electron transport chain, is known to interact with the coat proteins (CPs) of *Tomato mosaic virus* and *Cucumber mosaic virus*. However, our understanding of the roles of FD1 in virus infection remains limited. Here, we report that the *Potato virus X* (PVX) p25 protein interacts with FD1, whose mRNA and protein levels are reduced by PVX infection or by transient expression of p25. Silencing of *FD1* by *Tobacco rattle virus*-based virus-induced gene silencing (VIGS) promoted the local and systemic infection of plants by PVX. Use of a drop-and-see (DANS) assay and callose staining revealed that the permeability of plasmodesmata (PDs) was increased in *FD1*-silenced plants together with a consistently reduced level of PD callose deposition. After *FD1* silencing, quantitative reverse transcription–real-time PCR (qRT–PCR) analysis and LC-MS revealed these plants to have a low accumulation of the phytohormones abscisic acid (ABA) and salicylic acid (SA), which contributed to the decreased callose deposition at PDs. Overexpression of FD1 in transgenic plants manifested resistance to PVX infection, but the contents of ABA and SA, and the PD callose deposition were not increased in transgenic plants. Overexpression of FD1 interfered with the RNA silencing suppressor function of p25. These results demonstrate that interfering with FD1 function causes abnormal plant hormone-mediated antiviral processes and thus enhances PVX infection.

## Teaser

Interference with ferrodoxin 1 function results in low accumulation of callose at plasmodesmata and abnormal plant hormone-mediated antiviral processes, thus facilitating PVX infection.

## Introduction

There is increasing evidence that particular plant virus-encoded proteins directly target the chloroplast or interact with chloroplast proteins to facilitate viral infection or subvert the plant’s defense response (Zhao *et al.*, 2016; Bhattacharyya and Chakraborty, 2018). Numerous plant viruses locate their viral replication complexes (VRCs) to the double membrane of chloroplast to promote virus propagation while enabling newly synthesized dsRNA replication intermediates to evade detection by the host plant RNA silencing system (Prod’homme *et al.*, 2003; Dreher, 2004; Torrance *et al.*, 2006; Wei *et al.*, 2010, 2013). Some viral proteins possess chloroplast transit peptide (TP) domains to localize them to the chloroplast (Xiang *et al.*, 2006; Lim *et al.*, 2010; Bhattacharyya *et al.*, 2015), some viral proteins can themselves interact with the TP of chloroplast proteins (Qiao *et al.*, 2009), and others utilize unknown chloroplast targeting mechanisms (Torrance *et al.*, 2006; Xu and Zhou, 2012). Many new studies have now revealed interaction between photosynthesis electron transport chain-related proteins and plant virus proteins (Shi *et al.*, 2007; Cheng *et al.*, 2008; Lin *et al.*, 2011; Bhat *et al.*, 2013; Zhao *et al.*, 2013; Balasubramaniam *et al.*, 2014; Kong *et al.*, 2014; Chen *et al.*, 2018), although more research work needs to done to understand the outcomes of these interactions.

Ferredoxins (FDs), a category of small [2Fe–2S] cluster proteins, have roles in the chloroplast electron transport chain and also participate in the synthesis of chlorophyll, phytochrome, and fatty acids (Hanke and Mulo, 2013). There is increasing evidence that FDs and FD-like proteins are involved in interaction with plant pathogens (Huang *et al.*, 2007; Cheng *et al.*, 2008; Lin *et al.*, 2010; Sun *et al.*, 2013; Hou *et al.*, 2018; Qiu *et al.*, 2018; Wang *et al.*, 2018). For instance, the major leaf FD2, interacting with a harpin-binding protein FIBRILLIN4 (FIB4), regulates plant innate immunity in Arabidopsis against bacterial infection (Wang *et al.*, 2018). FD1 and FD5 have been shown to interact with coat protein (CP) of ToMV (*Tomato mosaic virus*), CMV (*Cucumber mosaic virus*), and SCMV (*Sugar cane mosaic virus*) HC-Pro, respectively (Cheng *et al.*, 2008; Sun *et al.*, 2013; Qiu *et al.*, 2018). Moreover, previous studies showed that the accumulation of FD1 was associated with development of the viral symptoms (Ma *et al.*, 2008; Hou *et al.*, 2018; Qiu *et al.*, 2018).

*Potato virus X* (PVX) is the type member of the genus *Potexvirus*, which is a worldwide group of economically important single-stranded positive sense RNA viruses (Adams *et al.*, 2004). The three PVX-encoded triple-gene-block (TGB) virus movement proteins (MPs) are involved in the spread of PVX within and between leaves (Krishnamurthy *et al.*, 2002; Morozov and Solovyev, 2003; Verchot-Lubicz *et al.*, 2007, 2010; Tilsner *et al.*, 2013). Among them, TGB1 (p25) has multiple functions during PVX infection. p25 modifies plasmodesmata (PDs), increases the PD size exclusion limit (SEL), forms a complex with TGB2, TGB3, and viral CP to assist viral cell to cell movement, and reorganizes the actin/endomembrane to contribute to PVX replication (Angell *et al.*, 1996; Yang *et al.*, 2000; Krishnamurthy *et al.*, 2002; Howard *et al.*, 2004; Tilsner *et al.*, 2012; Yan *et al.*, 2012). Moreover, p25 functions as an RNA helicase, a viral suppressor of RNA silencing (VSR), and a pathogenicity factor to trigger cell death in PVX-associated synergisms (Kalinina *et al.*, 2002; Chiu *et al.*, 2010; Aguilar *et al.*, 2015, 2019).

Modulation of the PD SEL is a factor that influences the ability of plant viruses to traffic between cells during plant virus infection, and is controlled by the deposition of callose, a linear β-1,3-glucan molecule, at the neck of the PDs (Bucher *et al.*, 2001; Iglesias and Meins, 2000; Fridborg *et al.*, 2003; Epel, 2009; Shemyakina *et al.*, 2011; Li *et al.*, 2012; Zavaliev *et al.*, 2013; Xiao *et al.*, 2018). The changes in the callose deposition level at the PDs is achieved by two mechanisms. Callose biosynthesis involves *callose synthase* (*CalS*) genes [or *glucan synthase-like* (*GSL*) genes], which are a group of 12 genes in Arabidopsis (Verma and Hong, 2001; Ellinger and Voigt, 2014). Among them, *CalS1*, *CalS8*, and *CalS10* in Arabidopsis have been proved to be involved in callose deposition at PDs, operating by different mechanisms (Guseman *et al.*, 2010; Ellinger and Voigt, 2014; Cui and Lee, 2016). The genes *CalS1* and *CalS8*, which are involved in PD callose deposition, are functionally dependent on salicylic acid (SA), whereas *CalS10* is not (Cui and Lee, 2016). Callose deposition is also controlled by the operation of β-1,3-glucanases, which can catalyse cleavage of callose into single β-1,3-glucan units (Doxey *et al.*, 2007). Previous studies have shown that the expression of β-1,3-glucanases is suppressed by the phytohormone abscisic acid (ABA), which increases callose PD deposition and decreases viral cell to cell movement (Rezzonico *et al.*, 1998; Iglesias and Meins, 2000; Bucher *et al.*, 2001; Levy *et al.*, 2007; Epel, 2009; H. Zhang *et al.*, 2015).

In this study, we demonstrate that PVX p25 interacts with the chloroplast protein FD1, and PVX infection or transient expression of p25 leads to low accumulation of FD1 in *Nicotiana benthamiana*. Silencing of *FD1* facilitates PVX cell to cell movement and replication. Drop-and-see (DANS) and callose staining assays show that in these plants PD callose deposition is significantly decreased, which is linked with a reduced accumulation of the phytohormones ABA and SA. Conversely, overexpression of FD1 interfered with the p25 silencing suppression function, by which PVX infection was reduced in FD1-overexpressing transgenic plants.

## Materials and methods

### Plant materials and inoculation of pathogens

Wild-type *N. benthamiana* and *FD1* transgenic *N. benthamiana* line OE FD1 (produced by Kai Yi Biotech Co., Ltd) were grown under a 16 h light/8 h dark regime at 25 °C. PVX was inoculated mechanically onto *N. benthamiana* according to the classical method (Li *et al.*, 2019), and Agrobacterium harbouring an infectious clone of PVX–GFP (green fluorescent protein) (Draghici *et al.*, 2009) was used for infiltration. GFP fluorescence was observed under long-wavelength UV-light (Black Ray Model B 100A, Ultra-Violet Products Ltd, Upland, CA, USA) and photographs were taken using a Cannon digital camera.

### *Agrobacterium* infiltration

For *Agrobacterium tumefaciens*-mediated transient expression, *Agrobacterium* strain GV3101 containing the expression vector was grown at 28 °C overnight, pelleted, re-suspended in infiltration buffer, and incubated at room temperature for 4 h. The *N. benthamiana* leaves were infiltrated with *A. tumefaciens* cultures with an OD_600_=0.5 and detached at 72 hours post-infiltration (hpi) for further analysis. For co-infiltrations, equal volumes of individual *Agrobacterium* cultures were mixed.

### Virus-induced gene silencing (VIGS)

*Tobacco rattle virus* (TRV) vectors, pYL196 (TRV1) and pTRV (TRV2), were kindly provided by Dr Yule Liu, Tsinghua University (Beijing, China) (Liu *et al.*, 2002). pTRV was used to silence host genes by expressing the partial sequence of different plant genes. To silence *FD1*, a 300 bp fragment of *FD1* was inserted into the pTRV vector, and an empty pTRV vector was used as control TRV:00. Primers used for the various constructions are listed in Supplementary Table S1 at *JXB* online. Silenced and control plants at 10 days post-infiltration (dpi) were used for further analysis.

### Total RNA extraction and RNA analysis

Total RNAs were isolated from leaves of *N. benthamiana* with Trizol (Invitrogen, USA) according to the manufacturer’s instructions. For quantitative reverse transcription–real-time PCR (qRT–PCR) of *FD1*, *N. benthamiana* Ubiquitin C (*UBC*) (AB026056.1) and *EF1A* (Niben101Scf08618g01002.1) were used as the internal reference genes for analysis. A Roche LightCycler^®^480 Real-Time PCR System was used for the reaction and the results were analysed by the ΔΔCT method. Similar methods were used to quantify expression of the callose-related genes *GLU1*, *Cals1*, and *Cals8*; ABA-related genes *ABA1*, *NCBD3*, *ABA2*, *AAO*, and *ABI1*; and SA-related genes *EDS1*, *ICS1*, *NPR1*, *PR1*, and *PDLP5*.

For northern blot analysis, a DNA probe targeting the PVX CP gene was synthesized and labelled with digoxin (DIG) according to the manufacturer’s protocol (DIG Oligonucleotide 3'-End Labelling Kit, Roche, Basel, Switzerland). Pre-hybridization, hybridization, and signal detection were performed according to the protocol of the DIG High Prime DNA Labelling and Detection Starter Kit II (Roche). Similar methods were used to detect endogenous *FD1* mRNA in *N. benthamiana*. Quantitative calculation of digital images of blots was done using ImageJ software.

### Western blotting

Total proteins of plant samples were extracted with lysis buffer (100 mM Tris–HCl pH 8.8, 60% SDS, 2% β-mercaptoethanol). Proteins were separated in 12% SDS–PAGE gels and detected with primary and secondary antibodies (Sigma-Aldrich, St. Louis, MO, USA). After incubation with secondary antibody, proteins were visualized with the EasySee Western Blot Kit (Transgene Biotech, BeiJing, China) and imaged with Molecular Imager ChemiDoc Touch (Bio-Rad). Quantitative calculation of digital images of blots was done using ImageJ software. The primary antibodies used in this research were anti-GFP (Transgene Biotech, BeiJing, China), and anti-FD1, anti-PVX p25, and anti-PVX CP, which were prepared in our laboratory.

### Yeast two-hybrid (Y2H) assays

Y2H analysis was performed following the Clontech yeast protocol handbook. The yeast expression vectors pGBK-p25, pGBK-FD1, pGAD-p25, and pGAD-FD1 were constructed. pGBK-p25/pGAD-FD1 were co-transformed into yeast cells. To confirm the successful co-transformation, the yeast cells were plated on a selective medium lacking tryptophan and leucine (SD/-Trp-Leu). Then, the transformed yeast cells were plated on deficient medium (lacking tryptophan, leucine, histidine, and adenine) supplemented with X-α-gal and Aureobasidin A (AbA) (Cerantola *et al.*, 2009), to analyse the interaction of the expressed proteins.

### Bimolecular fluorescence complementation (BiFC)

*FD1* was fused with the N- and C-terminal fragments of yellow fluorescent protein (YFP) to construct pYFPn-FD1 and pYFPc-FD1. p25 was fused with the N- and C-terminal fragments of YFP to construct pYFPn-p25 and pYFPc-p25. The plasmids were transformed into *A. tumefaciens* GV3101. For the BiFC assay, two agrobacteria cultures were mixed equally to OD_600_=0.1 and infiltrated into *N. benthamiana* leaves. At 48–72 hpi, the leaf tissues were observed under a Leica TCS SP5 confocal microscope (Leica Microsystems, Bannockburn, IL, USA). p25 self-interaction was used as a positive interaction control; GarVX TGB1 combined with p25 or FD1 were used as non-interacting paired controls.

### Co-immunoprecipitation (Co-IP)

pCV-FD1-GFP, pCV-p25, and pCV-eGFP were constructed and transformed into *A. tumefaciens* GV3101. Equal volumes of the agrobacteria suspension of pCV-FD1-GFP/pCV-p25 and pCV-eGFP/pCV-p25 were mixed (OD_600_=1.0) and infiltrated into *N. benthamiana* leaves. At 3 dpi, the leaves were collected and the native proteins were extracted. The protein extracts were incubated with GFP-Trap^®^_MA beads (Chromotek) according to the manufacturer’s instructions with some modifications. Briefly, ~0.2 g of leaf tissues was extracted with 0.5 ml of lysis buffer [10% glycerol, 25 mM Tris–HCl (pH 7.5), 1 mM EDTA, 150 mM NaCl, 2% (w/v) polyvinylpolypyrrolidone (PVPP), 10 mM DTT, 1× EDTA-free protease inhibitor cocktail (Roche), 1 mM phenylmethylsulfonyl fluoride (PMSF)]. After incubation on ice for 30 min, the mixtures were centrifuged at 10 000 g at 4 °C for 10 min. Extracts were incubated with GFP-Trap^®^_MA beads (ChromoTek) for 1 h at room temperature with gentle shaking. The beads were collected by brief centrifugation, washed four times with ice-cold lysis buffer at 4 °C, and analysed by immunoblotting with GFP or p25 antibodies.

### Leaf tissue dissection and observation

Leaves of plants were collected and immersed in primary fixation buffer (2% paraformaldehyde and 2.5% glutaraldehyde in 0.1 M potassium phosphate buffer, pH 6.8) overnight, followed by a secondary fixation with reduced osmium [1% OsO_4_ and 1.5% K_3_Fe(CN)_6_] and then a washing with 0.1 M potassium phosphate buffer. The fixed leaf samples were dehydrated by a series of ethanol/propylene oxide mixes and then embedded in Epon812 resin (Y. Zhang *et al.*, 2015). Cross-sections were stained by toluidine blue (Sakai, 1973) for 5 min and observed under a light microscope (Zeiss Axiovert A1, Carl Zeiss, Germany). At least 10 leaves from 10 individual plants were collected for detection of leaf thickness, which was analysed by ImageJ.

### Measurements of chlorophyll content

Tobacco leaf discs were put into a mixture of 96% acetone and ethanol at ratio of 2:1 and incubated in the dark for 24 h at 4 °C. The control solution was prepared with the same conditions without adding leaf discs. After removal of the leaf discs, the extracted solution was added to a 96-well plate with three technical replicates for each sample. Two absorption wavelengths of 663 nm and 645 nm were measured using a SpectraMax I3 (Molecular Devices, USA). Chl *a* and Chl *b* concentrations (mg l^–1^) were estimated using the following calculations: Chl *a* =12.7*A*_663_–2.69*A*_645_; Chl *b* =22.9*A*_645_–4.68*A*_663_.

### Protoplast extraction and determination of PVX replication in protoplasts

Protoplast extraction and plasmid transformation were done by using the plant protoplast preparation and transformation kit (RTU4052) [Real-Times (Beijing) Biotechnology Co., Ltd. The experiments were performed according to the manufacturer’s instructions. Briefly, the lower epidermis of leaves of TRV:FD1- and TRV:00-infiltrated plants were removed to expose the mesophyll cells. Then, the leaves were submerged in enzymatic hydrolysate solution 1 (supplied in the kit), 10 ml; cellulase R-10, 0.3 g; dissociation enzyme R-10, 0.08 g; reductant, 10 ml; 50 mg ml^–1^ BSA 0.4 ml) at room temperature for 3 h. The protoplasts were washed with solution 2 (supplied in the kit) and re-suspended in solution 3 (supplied in the kit). The number of protoplast cells was adjusted to 2×10^5^ cells per 100 ml. The PVX–GFP binary plasmid (10–20 μg) was added to the protoplasts which were incubated for 15 min at room temperature. The protoplasts were cultured at 27 °C and samples collected after 16 h and 24 h. Total RNA was extracted from the protoplasts using Trizol and the PVX RNA was measured by qRT–PCR.

### Quantitation of ABA and SA content in leaves by LC-MS

Leaves from TRV:FD1- and TRV:00-treated plants were harvested at 10 dpi. For ABA and SA quantification, ~50 mg of leaf tissue was finely ground in liquid nitrogen and extracted with 400 μl of 10% methanol containing 1% acetic acid to which internal standards had been added (1 ng of ^2^H_6_ ABA;13.8 ng of ^2^H_4_ SA). The quantification of ABA and SA was determined by LC-MS (Agilent 1260 Infinity-Agilent 6420A) as described previously (Yang *et al.*, 2019). These experiments were repeated three times, with each experiment containing three biological replicates. This work was done by Zoonbio Biotechnology Co., Ltd. The same method was used to analyse ABA and SA levels in OE 3.3 FD1-overexpressing plants and untreated, non-transgenic control plants.

### DANS assays

5(6)-Carboxyfluorescein diacetate (1 mM, CFDA) was used for a DANS dye-loading assay to analyse cell to cell movement (Cui *et al.*, 2015). Briefly, the dye was loaded as a 1 μl droplet onto a small puncture made in the central region in the upper leaf surface. After 30 min, the lower surface of the leaves was imaged by confocal microscopy. CFDA movement was measured as the fluorescent epidermal cell area surrounding the centre of loading. Each test was performed on 10 individual plants, and the dye diffusion diameter was analysed by ImageJ.

### Plasmodesmal callose staining

The callose in leaves was revealed following staining with 2 mg ml^–1^ aniline blue (Biosupplies). Aniline blue was dissolved in sodium phosphate buffer, pH 7.5, injected into the epidermal cells of leaves, and incubated for 5 min in the dark. Then, the injected leaf tissue was dissected out, washed with sterile water, and observed under a Leica TCS SP5 confocal microscope (Leica Microsystems, Bannockburn, IL, USA).

### ABA and SA treatments

The *N. benthamiana* plants were sprayed daily with ABA (Sigma, A1049) at a final concentration of 100 μM for 3 d with ddH_2_O as a control. Other plants were sprayed daily with 1 mM SA dissolved in 0.1% (v/v) ethanol for 3 d. Mock control plants were treated with 0.1% ethanol. ABA or SA was sprayed on both adaxial and abaxial sides until the liquid ran off the leaves (Alazem *et al.*, 2017). All treatments were performed independently at least three times and at least three sets of consistent data were used for further analysis.

## Results

### PVX p25 interacts with the chloroplast protein FD1

**Fig. 1.**
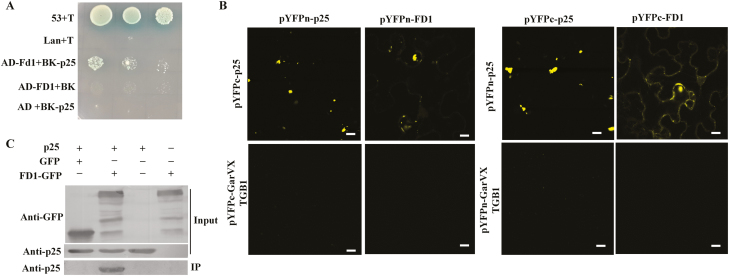
FD1 interacts with p25. (A) Interaction between FD1 and p25 was confirmed by yeast co-transformation with the plasmids shown on the left side of the panel and growth on an SD/-Leu/-Trp/-His/-Ade/X-α-Gal/AbA medium. (B) Interaction between FD1 and p25 revealed by BiFC in agrofiltrated *N. benthamiana* leaves. Scale bars=25 μm. (C) Interaction between FD1 and p25 verified by co-immunoprecipitation. The input proteins were detected by western blotting with anti-FD1 and anti-p25 antibodies. IP denotes the immunoprecipitated fraction probed with anti-p25 antibodies.

A Y2H screen was performed of an *N. benthamiana* cDNA library to identify plant proteins that interact with PVX p25. Under stringent selective conditions, a candidate p25-interacting protein, FD1 (KP763813.1), was identified and selected for further investigation. The complete ORF of FD1 was amplified from *N. benthamiana* cDNA and cloned into pGEM-T for sequencing and further manipulations. FD1, fused at the C-terminus with GFP or red fluorescent protein (RFP), was transiently expressed in *N. benthamiana* leaves and shown by confocal microscopy to localize to the chloroplast (Supplementary Fig. S1). The complete ORF of FD1 was cloned into pGADT7 to verify the Y2H interaction between FD1 and p25 (Fig. 1A).Yeast growth occurred on nutrient-deficient, selective medium when FD1 [fused to the Gal4 activation domain (AD)] and p25 [fused to the Gal4 DNA-binding domain (BD)] were co-transformed into cells, but no growth occurred when either FD1 or p25 were co-transformed with empty BD or AD domain plasmids.

To confirm that this interaction also occurred in plants, we performed BiFC experiments by transient expression of paired proteins in *N. benthamiana* leaves. In this assay, FD1 and p25 were fused at the C-terminus of either the amino (n) domain or the carboxy (c) domain of YFP (Lu *et al.*, 2011; Yan *et al.*, 2012), respectively, to generate constructs pCV-YFPn-FD1, pCV-YFPc-FD1, pCV-YFPn-p25, and pCV-YFPc-p25, with the GarVX (*Garlic virus X*) TGB1 protein used as a non-interacting control. Co-expression of pCV-YFPn-FD1 and pCV-YFPc-p25 or of pCV-YFPc-FD1 and pCV-YFPn-p25 resulted in YFP fluorescence signals in the cytoplasm of agro-infiltrated cells at 72 hpi, demonstrating interaction between these two proteins (Fig. 1B). The known self-interaction of p25 confirmed that the BiFC system was functioning as expected (Fig. 1B), and the lack of fluorescence demonstrating no interaction between p25 and GarVX-TGB1 or FD1 and GarVX-TGB1 showed that neither FD1 nor p25 could produce YFP fluorescence in the absence of a bona fide interacting partner (Fig. 1B).

In addition, a Co-IP assay was performed to further verify the interaction between FD1 and p25. In this assay, plasmids encoding FD1–GFP and p25 were transiently expressed in *N. benthamiana*, with unfused GFP being used as a negative control, and GFP-TRAP_M beads being used to collect GFP-containing proteins and any interacting partners accumulating in the plants. Although expression of all the proteins was confirmed in the plants (Fig. 1C, input), p25 was co-precipitated only in the presence of FD1–GFP and not when expressed alone or together with unfused GFP.

### PVX infection or transient expression of p25 results in low accumulation of FD1

**Fig. 2.**
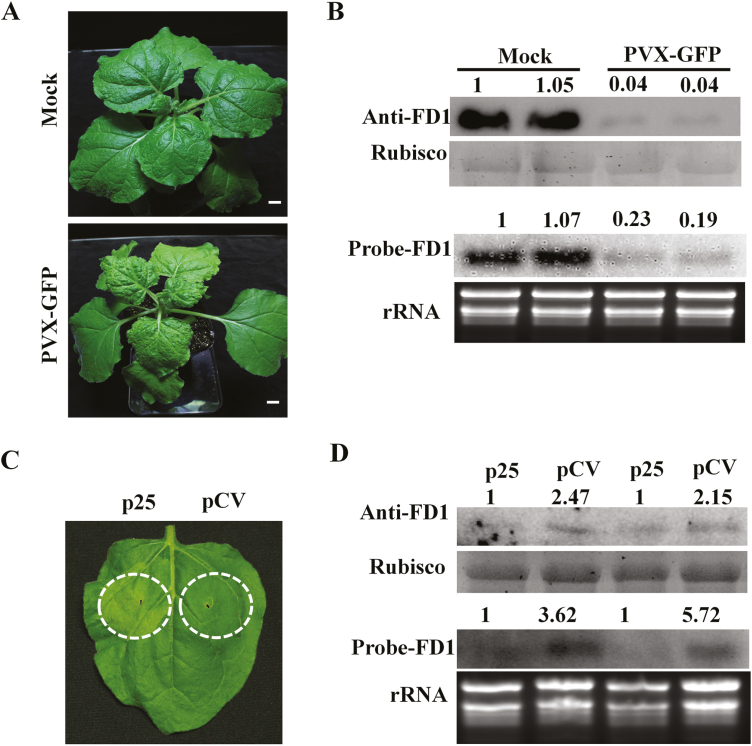
PVX and p25 reduce the accumulation of FD1. (A) *N. benthamiana* leaves were inoculated with an extract of a PVX–GFP-infected plant or a healthy plant as a mock treatment and photographed at 9 dpi. Scale bars represent 2 cm. (B) The FD1 protein accumulation in PVX-infected and mock-inculated leaves was detected by western blotting with an anti-FD1 antibody. Rubisco was used as a loading control. (C) The mRNA accumulation of *FD1* in PVX-infected and mock-inoculated leaves was detected by northern blot analysis with a probe to *FD1*. rRNA was used as a loading control. (D) *N. benthamiana* leaves were infiltrated with agrobacteria harbouring pCV-p25 and pCV plasmids, and photographed at 3 dpi. (E) The accumulation of FD1 protein in p25- and pCV-infiltrated leaves was detected by western blot analysis with an anti-FD1 antibody. (F) The mRNA accumulation of *FD1* in p25- and pCV-expressing leaves was detected by northern blot analysis with a probe to *FD1*. The relative intensities of the blot signals were quantified by ImageJ as shown above the lanes.

Because of the interaction between p25 and FD1, we theorized that FD1 was involved in some part of the PVX infection process. To further investigate the details, the levels of FD1 protein and mRNA in PVX-infected plants were assayed by qRT–PCR, western blotting, and northern blotting. At 9 dpi with PVX expressing the GFP gene (PVX–GFP), typical yellow mosaic and leaf curling symptoms developed in the upper, systemically infected leaves (Fig. 2A). The results of qRT–PCR and northern blotting showed that *FD1* mRNA accumulation was reduced by >50% in PVX-infected plants compared with mock-inoculated plants (Fig. 2C; Supplementary Fig. S2A). Furthermore, the FD1 protein content was reduced by 95% in systemic leaves of the PVX-infected plants (Fig. 2B), which was consistent with the reduction in *FD1* mRNA.

Similar experiments were done to examine the accumulation of FD1 in leaves in which the p25 protein was transiently expressed, comparing these with leaves in which an empty expression vector (pCV) was infiltrated (Fig. 2D). At 3 dpi, qRT–PCR analysis showed that the transcription level of *FD1* was reduced by 50% in leaves treated with p25 compared with control leaves (Supplementary Fig. S2B). FD1 protein levels were >2-fold higher in control tissue compared with p25-containing tissue, and by northern blotting *FD1* mRNA levels were found to be >3-fold higher in control tissue compared with p25-containing tissue (Fig. 2E, F). All these results indicate that *FD1* mRNA and protein accumulation are significantly reduced by PVX infection or by expression of p25.

### Silencing of *FD1* promotes PVX systemic infection in *N. benthamiana*

To further characterize the involvement of FD1 in the infection of PVX, we used the TRV VIGS system to silence *FD1* in *N. benthamiana* plants before inoculation with PVX–GFP. At 10 dpi, the *FD1*-silenced plants (TRV:FD1) had chlorotic upper leaves whereas the TRV:00-treated plants remained green (Supplementary Fig. S3A). qRT–PCR showed that the transcript level of *FD1* in TRV:FD1-silenced plants was only 2% of that in TRV:00-treated plants (Supplementary Fig. S3A). This was accompanied by a reduction in chlorophyll content of ~75% in *FD1*-silenced plants (Supplementary Fig. S3B). Light microscopy of leaf tissue slices showed that the thickness of leaves in *FD1*-silenced plants was reduced to 65% of that in non-silenced plants (Supplementary Fig. S3C). Furthermore, we found that the dry weight/wet weight ratio of TRV:FD1 leaves decreased by 20% compared with TRV:00 leaves (Supplementary Fig. S3D).

**Fig. 3.**
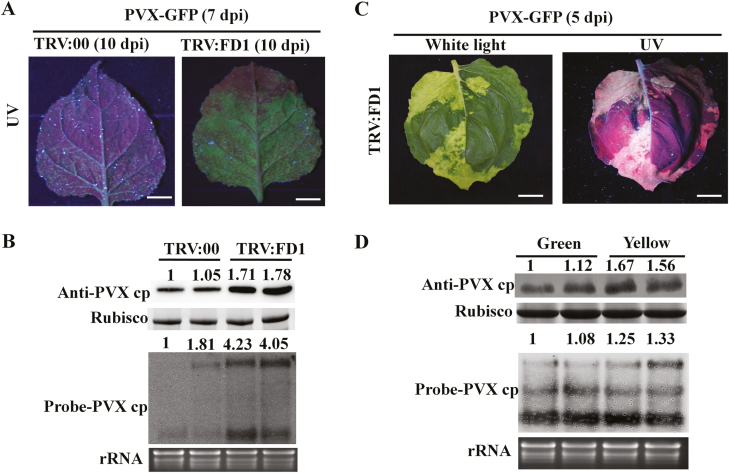
Silencing of *FD1* promotes PVX accumulation. (A) Leaves treated with TRV:00 and TRV:FD1 at 10 dpi were secondarily infected with PVX–GFP. Pictures of systemically infected leaves taken at 7 dpi with PVX–GFP under UV light. Scale bars represent 1 cm. (B) PVX–GFP accumulation in systemically infected leaves (7 dpi) was examined by western blot analysis with PVX CP antibody and northern blot analysis with a probe to PVX CP RNA. (C) Leaves of *FD1-*silenced plants infected with PVX–GFP and displaying sectoring, pictured at 5 dpi. Scale bars represent 1 cm. (D) PVX–GFP accumulation in dark green or yellow regions of TRV:FD1-silenced leaves (5 dpi) was examined by western and northern blot analysis. The relative intensities of the blot signals were quantified by ImageJ as shown above the lanes.

At 10 dpi with TRV:FD1 or TRV:00, the plants were again infiltrated with agrobacteria harbouring PVX–GFP. The progress of viral infection into the top-most leaves was monitored with a UV lamp to reveal the location and extent of GFP fluorescence produced by PVX. The development index of PVX–GFP symptom production in plants pre-treated with TRV:FD1 and TRV:00 is presented in Supplementary Fig. S4A. All TRV:FD1 plants had systemic PVX infection at 6 dpi, whereas the TRV:00 plants became 100% systemically infected 1 d later. In addition, the GFP fluorescence was stronger in the upper leaves of TRV:FD1 plants compared with the TRV:00 plants (Fig. 3A). Western and northern blotting results showed higher levels of PVX CP mRNA and protein in *FD1*-silenced plants than in non-silenced plants, which was consistent with the brighter fluorescence in TRV:FD1-treated plants (Fig. 3B). In some plants, the systemic leaves of TRV:FD1 plants had both green and yellow areas in the same leaf (Fig. 3C left). qRT–PCR analysis showed that the silencing of *FD1* was more effective in the yellow areas than in the green areas (Supplementary Fig. S4B). In addition, both PVX CP mRNA and protein accumulation levels were higher in the yellow areas than in the green areas (Fig. 3D). Taken together, these results suggest that silencing of *FD1* facilitates the systemic infection of PVX in *N. benthamiana*.

### Silencing of *FD1* promotes local infection by PVX

The experiments described so far involve an examination of the influence of FD1 on systemic infection by PVX, which could be controlled by differences in the passage of virus from lower to upper leaves via the vasculature. We therefore performed more experiments to see whether FD1 could influence PVX infection in the initial infected leaf, by affecting the rate of virus replication (increase in titre in an individual cell) and/or by altering the rate at which virus could move between adjacent cells in the same leaf.

**Fig. 4.**
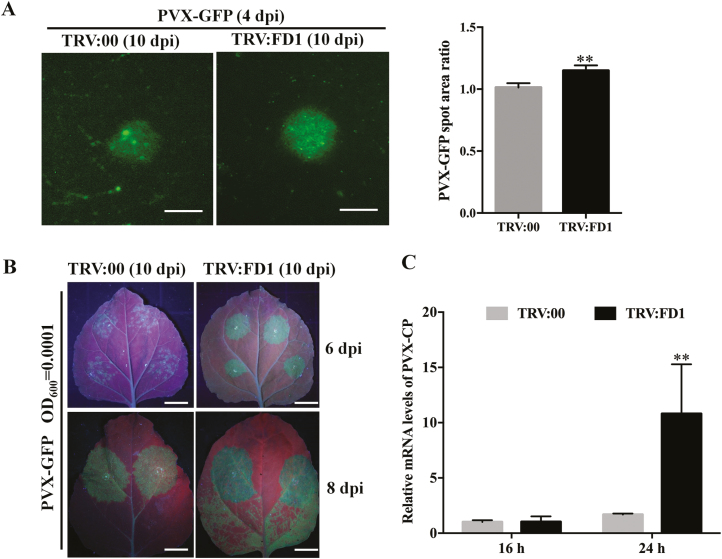
Silencing of *FD1* increases intercellular movement and replication of PVX. (A) PVX–GFP fluorescent spots on inoculated leaves of TRV:00- and TRV:FD1-treated plants were detected using an inverted fluorescence microscope. The area of PVX–GFP spots was measured using ImageJ software. Scale bars=1 mm. (B) Pictures of PVX–GFP-infiltrated leaves taken at 6 and 8 dpi under UV light. Leaves of TRV:00 and TRV:FD1 at 10 dpi were secondarily infected with an *Agrobacterium* culture of PVX–GFP (OD_600_=0.0001). Scale bars represent 1 cm. (C) The accumulation of PVX CP mRNA was measured by qRT–PCR in PVX-infected protoplasts generated from TRV:00- or TRV:FD1-treated *N. benthamiana* leaves after 16 h and 24 h incubation. Bars represent the SE of the means from three biological repeats. A two-sample unequal variance directional *t*-test was used to test the significance of the difference (*P-value <0.05; **P-value <0.01).

The plants treated with TRV:FD1 or TRV:00 were mechanically inoculated with an extract of *N. benthamiana* leaves containing infectious PVX–GFP, so that the establishment and development of individual infection foci could be observed. At 4 dpi, fluorescent spots of PVX–GFP infection became apparent in the inoculated leaves. The size of the spots increases as PVX–GFP moves laterally from cell to cell. Using a fluorescence microscope, we found that the area of fluorescent spots on TRV:FD1-treated plants was statistically significantly larger than that on control TRV:00-treated plants (Fig. 4A). In a similar experiment, *FD1*-silenced and non-silenced control plants were agro-infiltrated with PVX–GFP at a very low bacterial concentration (OD_600_=0.0001). At 6 dpi, visibly separate green fluorescent infection foci were distributed in the infiltrated patches of TRV:00-treated plants, whereas the infiltrated patches of TRV:FD1-treated plants were completely filled with a uniform area of fluorescence (Fig. 4B upper panels). At 8 dpi, in *FD1*-silenced plants, the green fluorescence had moved out of the infiltrated patches and into adjacent tissue. However, the green fluorescence was limited to inside the infiltrated patches of non-silenced plants (Fig. 4B bottom panels).

The binary plasmid encoding infectious PVX–GFP was transfected into protoplasts isolated from either *FD1*-silenced plants or non-silenced control plants. qRT–PCR results showed that after 24 h incubation the accumulation of PVX RNA in *FD1*-silenced protoplasts was nearly 10-fold higher than in control plants (Fig. 4C). These data suggest that silencing of *FD1* facilitates the viral cell to cell movement and replication in local infection.

### The deposition of PD callose is altered in *FD1*-silenced plants

**Fig. 5.**
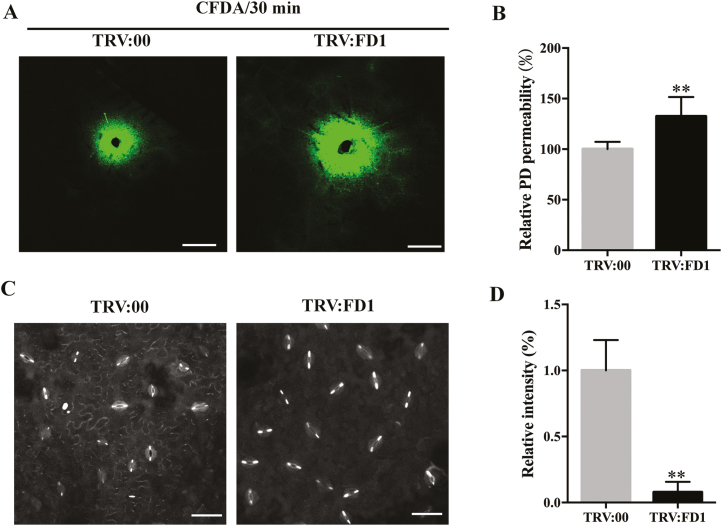
Silencing of *FD1* reduces the accumulation of PD callose. (A) DANS assays on TRV:00- and TRV:FD1-treated leaves showing cell to cell diffusion of 5(6)-carboxyfluorescein diacetate (CFDA); scale bars=500 μm. (B) The PD permeability was quantitated using ImageJ software. (C, D) Aniline blue staining of TRV:00- and TRV:FD1-treated leaves revealed the callose in PDs and guard cells. Scale bars=50 μm. In (D), bars represent the SE of the means from three biological repeats. A two-sample unequal variance directional *t*-test was used to test the significance of the difference (*P-value <0.05; **P-value<0.01).

PDs are lipid and protein channels that traverse the cell wall to connect adjacent cells. Plant viruses use these channels to spread outwards from a point of initial infection, in an active process referred to as cell to cell movement. The SEL, which describes the maximum size of molecule that can freely diffuse through an individual PD, is increased by some virus-encoded proteins to facilitate the cell to cell movement of viruses during infection (Heinlein, 2015). Since the cell to cell movement of PVX was enhanced in *FD1*-silenced plants, we investigated whether the permeability of PD was different in *FD1*-silenced plants and non-silenced plants. We used the DANS assay (Cui *et al.*, 2015) to detect the diffusion rate of CFDA between cells, which reflects the permeability of PDs. We added the same volume of CFDA (1 mM) on leaves of both TRV:FD1- and TRV:00-treated plants, and the extent to which the dye spread laterally between cells was observed by confocal microscopy and measured. The results showed that the range and speed of CFDA diffusion increased significantly in leaves of TRV:FD1-treated plants compared with those in control plants at 30 min (Fig. 5A, B).

The permeability of PDs is directly correlated with the extent of callose deposition within them. Quantitative analysis of aniline blue staining of PD callose revealed that there was a very low amount of callose deposited on the PDs in leaves of TRV:FD1-treated plants, which was only 7% of the PD callose existing in the leaves of TRV:00-treated, control plants (Fig. 5C, D). To understand the very low deposition of PD callose in *FD1*-silenced leaves, we used qRT–PCR to analyse the RNA expression level of β-1,3-glucanase (GLU1), CalS1, and CalS8, enzymes which either negatively or positively regulate the deposition of PD callose (Bucher *et al.*, 2001; Cui and Lee, 2016). The results showed that the transcript level of *GLU1* in *FD1*-silenced plants was significantly increased compared with that in non-silenced plants (Supplementary Fig. S5A), and the mRNA level of *Cals1* was reduced in *FD1*-silenced plants compared with that in non-silenced plants, whereas there was no difference in the *Cals8* transcript level in either plant (Supplementary Fig. S5B). An increase in *GLU1* and a decrease in *Cals1* could both contribute to reducing the amount of PD callose in *FD1*-silenced plants.

### Down-regulation of ABA and SA resulted in low deposition of PD callose in *FD1*-silenced plants

**Fig. 6.**
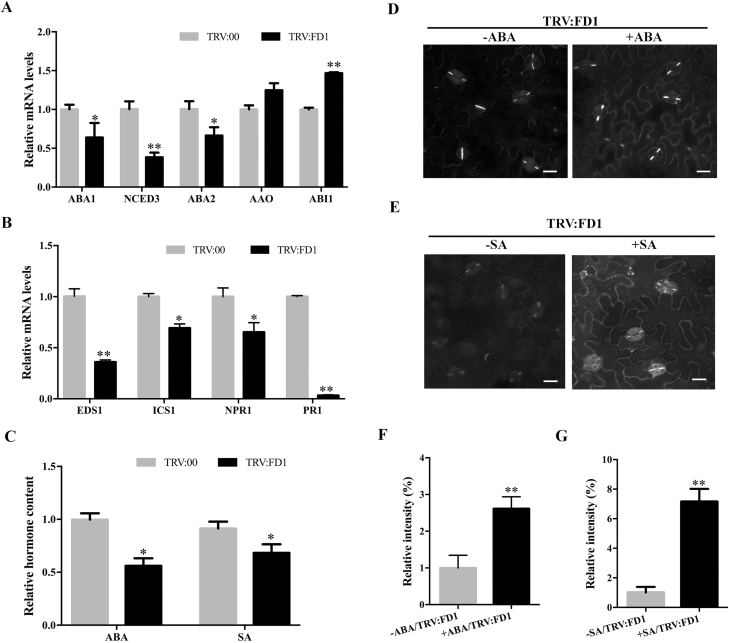
The deposition of PD callose in TRV:FD1-treated plants is associated with accumulation of ABA and SA. (A, B) qRT–PCR was used to examine the transcript levels of ABA pathway-related genes *ABA1*, *NCBD3*, *ABA2*, *AAO*, and *ABI1*; and SA-dependent signaling pathway-related genes *EDS1*, *ICS1*, *NPR1*, *PR1*, and *PDLP5* of TRV:00- and TRV:FD1-treated plants. (C) The relative levels of ABA and SA in TRV:FD1- and TRV:00-treated plants were measured by LC-MS. (D, F) TRV:FD1-treated plants were sprayed with 100 μM ABA or ddH_2_O, and (E, G) 1 mM SA or 0.1% (v/v) ethanol. Callose accumulation was revealed by aniline blue staining. Bars represent the SE of the means from three biological repeats. A two-sample unequal variance directional *t*-test was used to test the significance of the difference (*P-value <0.05; **P-value <0.01).

Phytohormones participate in the endogenous immune system in plants and influence their resistance to plant diseases. The phytohormones ABA and SA are both reported to regulate the deposition of PD callose (Mauch-Mani and Mauch, 2005; Cui and Lee, 2016). SA can induce the synthesis of endogenous PD callose in plants by up-regulating *Cals1* and *Cals8* (Cui and Lee, 2016). To determine whether SA and ABA are involved in the alteration in PD callose deposition observed in our work, we analysed the transcription levels of genes involved in ABA and SA synthesis and signalling pathways by qRT–PCR comparing *FD1*-silenced and non-silenced plants. For ABA pathway analysis, we selected the genes *ABA1* (ABA deficient 1, which encodes zeaxanthin epoxidase), *NCBD3* (9-*cis*-epoxycarotenoid dioxygenase 3), *ABA2* (ABA deficient 2, which encodes xanthoxin dehydrogenase), *AAO3* (abscisic aldehyde oxidase 3), and *ABI1* (ABA-insensitive 1) (Alazem *et al.*, 2014). The mRNA levels of the ABA synthesis-related genes *ABA1*, *NCBD3*, and *ABA2* were reduced in *FD1*-silenced leaves compared with non-silenced leaves (Fig. 6A), while the mRNA level of *ABI1* (Merlot *et al.*, 2001), which negatively regulates the accumulation of ABA, was significantly increased in TRV:FD1-treated plants (Fig. 6A). Also, the transcription levels of the SA synthesis genes *EDS1* (enhanced disease susceptibility 1) and *ICS1* (isochorismate synthase 1), and the SA-dependent signaling-related genes, *NPR1* (nonexpressor of pathogenesis related genes 1) and *PR1* (nathogenesis-related protein 1) were significantly reduced in *FD1*-silenced leaves compared with non-silenced leaves (Fig. 6B).

To confirm the down-regulation of ABA and SA biosynthetic pathways in *FD1*-silenced plants, we used LC-MS to measure the amounts of ABA and SA in TRV:FD1- and TRV:00-treated plants. This analysis showed that the ABA content was nearly 50% lower and the SA content nearly 40% lower in TRV:FD1-silenced plants compared with TRV:00-treated control plants (Fig. 6C).

To investigate whether synthesis of ABA and SA was specifically affected by reduction in FD1 expression or whether changes in the expression of other chloroplast genes would have similar effects, we used VIGS to silence four other chloroplast genes, ferredoxin-NADP (H) oxidoreductase (*FNR*), chloroplast oxygen-evolving protein 33 kDa subunit (*psbO*), chloroplast oxygen-evolving protein 17 kDa subunit (*psbQ*), and ribulose-1,5-bisphosphate carboxylase/oxygenase activase (*RCA*), in *N. benthamiana* plants (Supplementary Fig. S6). The mRNA levels of ABA- and SA-dependent pathway-related genes were not down-regulated in TRV:RCA- or TRV:psbQ-silenced plants (Supplementary Figs S7, S8), although *EDS1* expression was increased in *FNR*-, *RCA1*-, and *PsbQ*-silenced plants. Also, silencing of *FNR* did reduce expression of *ABA1*, *ABA2*, and *AAO3*, and silencing of *PsbO* did reduce expression of *NCED3*, *ABA2*, and *AAO3*. Interestingly, *ABA1* expression was reduced by *FNR* silencing but increased by *PsbO* silencing. All these data indicated that ABA and SA gene expression is influenced by FD1 and several other chloroplast genes, although the relationship between the different genes is complex and not clearly defined.

To investigate whether there is a direct relationship between *FD1* gene expression, phytohormone accumulation, and PD callose deposition, we determined whether treatment of *FD1*-silenced plants with exogenous ABA or SA would correct the previously observed reduction in PD callose levels, In these experiments, TRV:FD1-silenced plants were treated daily by spraying with either 100 μM ABA, 1 mM SA, ddH_2_O (as the control for ABA), or 0.1% ethanol (as the control for SA). Quantitative analyses of aniline blue-stained PD callose 4 d after the start of the treatments revealed that the deposition of PD callose in the ABA-treated and SA-treated leaves of *FD1*-silenced plants was increased 2.5- and 7-fold, respectively, compared with leaves from *FD1*-silenced plants that had been sprayed with control treatments (Fig. 6D–G). In *FD1*-silenced plants, the transcription level of *GLU1* in ABA-treated leaves was decreased by 50% compared with mock-treated leaves (Supplementary Fig. S9A), whereas expression of the PD callose synthesis-related gene *Cals1* was increased by 50% after treatment with SA (Supplementary Fig. S9B). These data collectively suggest that silencing of *FD1* leads to a decrease in the accumulation of ABA and SA that, subsequently, leads to a reduction in PD callose deposition.

### Overexpression of FD1 increased the resistance of PVX in *N. benthamiana*

**Fig. 7.**
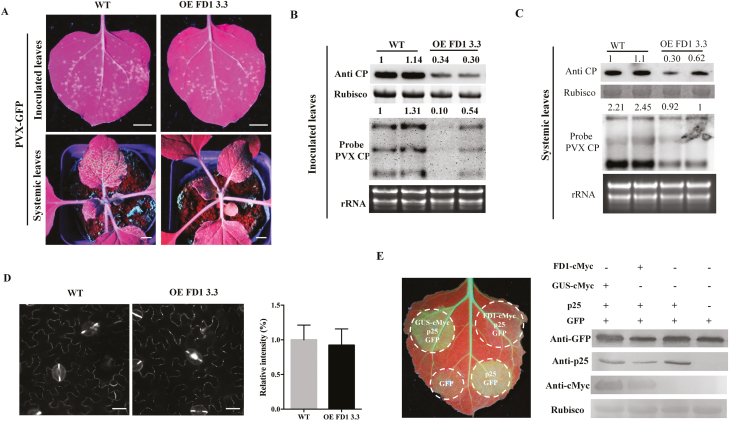
Overexpression of FD1 in *N. benthamiana* reduces accumulation of PVX. (A) Pictures of inoculated leaves and systemic leaves of OE FD1 3.3 and wild-type (WT) plants infected with PVX–GFP viewed under UV light. Scale bars represents 1 cm. The PVX CP protein and mRNA accumulation in OE FD1 3.3 and the WT inoculated (B) and systemic (C) leaves were analysed by western blot and northern blot with an anti-PVX CP antibody and a probe to PVX CP mRNA. (D) Callose deposition in WT and OE FD1 3.3 leaves. (E) Silencing suppression ability of p25 was tested in GFP-transgenic *N. benthamiana* plants (16c), with transient co-expression of GFP and FD1 or GUS (as a control protein). GFP fluorescence was revealed by UV illumination at 5 d post-infiltration with *Agrobacterium* constructs. Bars represent the SE of the means from three biological repeats. A two-sample unequal variance directional *t*-test was used to test the significance of the difference (*P-value <0.05; **P-value <0.01).

To further investigate the potential function of FD1 against PVX infection, we made stable transgenic lines of *N. benthamiana* overexpressing FD1 and analysed the infection of PVX on these plants. The full-length *FD1* gene driven by the *Cauliflower mosaic virus* (CaMV) 35S promoter was overexpressed in plants through *Agrobacterium*-mediated transformation. Three independent transgenic lines (lines OE 3.3, OE 9.3, and OE 13.2) with up-regulated expression of *FD1* were chosen for analysis with qRT–PCR and western blotting (Supplementary Fig. S10A, B). All lines developed normally without any obviously changed phenotype. Green fluorescent spots and PVX CP accumulation were measured on inoculated leaves of wild-type, OE 3.3, OE 9.3, and OE 13.2 plants (Supplementary Fig. S10C–E). All three OE lines had reduced numbers and diameter of PVX–GFP infection foci compared with wild-type plants, and both OE lines 3.3 and 13.2 had reduced accumulation of PVX CP. Thereafter, line OE 3.3 was used to analyse local and systemic infection of PVX, compared with wild-type (non-transgenic) plants. At 4 dpi, green fluorescent spots became visible on both OE 3.3- and wild-type-inoculated leaves (Fig. 7A, upper panels). Western blot and northern blot results showed that the accumulation of PVX CP protein and mRNA in OE 3.3-inoculated leaves was reduced compared with that in wild-type plants (Fig. 7B). At 6 dpi, the intensity of green fluorescence was brighter in OE 3.3 systemic leaves compared with wild-type plants (Fig. 7A, lower panels). As shown in Fig. 7C, the accumulation of PVX CP protein and mRNA in OE 3.3 systemic infected leaves was reduced compared with those in wild-type plants.

To explore whether resistance of PVX in OE 3.3 plants was related to the deposition of PD callose, quantitative analysis of aniline blue-stained PD callose was carried out. As shown in Fig. 7D, there was no difference in deposition of PD callose between OE 3.3 leaves and wild-type leaves. Furthermore, the endogenous ABA and SA levels were similar in OE 3.3 and wild-type plants (Supplementary Fig. S10F). These results indicate that the improved resistance of OE 3.3 plants against PVX was not associated with either increased callose deposition or increased ABA and SA content. Since FD1 interacted with p25 (Fig. 1), we hypothesized that the overexpression of FD1 might interfere with the function of p25 as a suppressor of RNA silencing that is essential for viral infection. To test this, the silencing suppression activity of p25 was examined in GFP-transgenic *N. benthamiana* plants (16c). In these experiments, silencing of the GFP transgene was initiated by infiltration with an *Agrobacterium* culture carrying a second copy of the GFP gene. As shown in Fig. 7E, at 5 dpi, the amount of GFP fluorescence was higher in leaf patches co-infiltrated with plasmids expressing GFP and p25, and also GFP and p25 plus GUS–cMyc (used as an internal control) compared with a patch co-infiltrated with the GFP plasmid only. These results demonstrated that p25 was able to suppress silencing of the GFP gene in these infiltrated patches. In contrast, GFP fluorescence was much lower in a patch co-infiltrated with GFP and p25 plus FD1–cMyc, indicating that the FD1–Myc could prevent p25 from suppressing silencing of the GFP gene. Western blotting was done to confirm that the levels of apparent GFP fluorescence correctly reflected the level of GFP protein accumulation in the various patches (Fig. 7E). These results support the notion that the suppression of PVX infection in OE 3.3 plants could result from an interference with the p25 RNA silencing suppression function by FD1.

## Discussion

Numerous recent studies using proteomic and transcriptomic approaches have shown that plant viral infections cause alteration in the expression of chloroplast- and photosynthesis-related genes (Wu *et al.*, 2013; Liu *et al.*, 2014; Mochizuki *et al.*, 2014). Plant viruses or viral proteins target chloroplast proteins to achieve two aims: (i) manipulating chloroplast proteins to aid viral infection (Harries *et al.*, 2009); and (ii) disturbing the function of chloroplast proteins to facilitate the viral infection in plants (Zhao *et al.*, 2013; Balasubramaniam *et al.*, 2014; Kong *et al.*, 2014; DeBlasio *et al.*, 2018). In this study, we identified a chloroplast protein, FD1, which was targeted by PVX p25, and revealed several mechanisms promoting PVX infection in *FD1-*silenced plants.

In the chloroplast, FD1 acts as an electron carrier in the photosynthetic electron transport chain and also as an electron donor for many proteins such as glutamate synthase (Suzuki *et al.*, 1988), nitrite reductase (Terauchi *et al.*, 2009), and sulfite reductase (Saitoh *et al.*, 2006). Previous reports have demonstrated that FD1 is involved in the development of viral symptoms (Ma *et al.*, 2008; Hou *et al.*, 2018; Qiu *et al.*, 2018). Moreover, FD1 can directly interact with several virus-encoded proteins (Sun *et al.*, 2013; Qiu *et al.*, 2018).

A number of host factors, including two chloroplast-localized proteins AtNHR2A and AtNHR2B, are reported to regulate callose deposition during plant disease (Fridborg *et al.*, 2003; Oide *et al.*, 2013; Seo *et al.*, 2014; Pillai *et al.*, 2018; Singh *et al.*, 2018). Callose deposition at PDs directly affects its SEL and permeability (Cui and Lee, 2016), which is linked to the cell to cell movement of *Tobacco mosaic virus* (TMV), *Soybean mosaic virus* (SMV), *Melon necrotic spot virus* (MNSV), and PVX (Fridborg *et al.*, 2003; Serova *et al.*, 2006; Li *et al.*, 2012; Fernández-Crespo *et al*., 2017; Xiao *et al.*, 2018). Here, we found that the cell to cell movement of PVX was promoted in *FD1-*silenced plants, which also manifested a low level of PD callose deposition (Fig. 5). Although deposition of PD callose was significantly decreased in *FD1-*silenced plants, a similar effect was not seen in transgenic plants overexpressing FD1 (Fig. 7D, E). Hence, we suggested that the deposition of PD callose is not directly regulated by FD1.

The phytohormones ABA and SA play important roles in plant development and response to stress, and also participate in the regulation of callose deposition (Oide *et al.*, 2013; Cui and Lee, 2016). In addition, redox homeostasis and calcium are involved in regulating callose deposition at PDs (Holdaway-Clarke *et al.*, 2000; Benitez-Alfonso and Jackson, 2009, 2011; Stonebloom *et al.*, 2012). ABA treatment can interfere with the infection process of several viruses, for example TMV (Whenham *et al.*, 1986), *Tobacco necrosis virus* (Iriti and Faoro, 2008), *Bamboo mosaic virus* (Alazem *et al.*, 2014), and SMV (Li *et al.*, 2012; Seo *et al.*, 2014), by causing deposition of callose at PDs and inducing the expression of RNA silencing-related genes. It is reported that SA treatment induces callose deposition at PDs to restrict their permeability, which also involves NPR1 and plasmodesmal localization protein 5 (PDLP5) (Wang *et al.*, 2013; Cui and Lee, 2016). PDLP5, induced by SA, controls the expression of *CalS1* and *CalS8* in Arabidopsis, which are responsible for the synthesis and deposition of callose at PDs. Here, we found that silencing *FD1* did decrease the levels of ABA and SA (Fig. 6C). Furthermore, exogenous addition of ABA and SA could significantly increase the PD callose deposition in *FD1-*silenced plants (Fig. 6D–G), indicating that the reduction of PD callose deposition in *FD1-*silenced plants was related to low accumulation of the phytohormones ABA and SA.

The biosynthesis of ABA and SA normally occurs in the chloroplast, which is also the site of localization of FD1. However, a recent report shows that pathogen-induced SA synthesis derived from isochorismate is generated in the cytoplasm in Arabidopsis (Rekhter *et al.*, 2019). Similarly, it was reported that disruption of the chloroplast gene phytoene desaturase (*PDS*) impaired biosynthesis of another phytohormone, gibberellic acid (GA) (Qin *et al.*, 2007). We reduced the expression individually of four other chloroplast genes, *FNR*, *psbO*, *psbQ*, and *RCA*, and showed that, in some cases, this affected the mRNA levels of several ABA- and SA-dependent signalling pathway-related genes (Supplementary Figs S7, S8). The results indicated that the relationship between these chloroplast genes and SA and ABA biosynthesis is complex and not yet clearly defined. Previous research found that in Arabidopsis ectopic expression of ferredoxin-like proteins enhanced resistance to bacterial pathogens (Lin *et al.*, 2010; Ger *et al.*, 2014). Here, our results demonstrated that in transgenic *N. benthamiana*, overexpression of FD1 increased resistance to PVX infection (Fig. 7A–C). However, overexpressed FD1 did not increase the levels of ABA, SA, and the PD callose, which were dramatically decreased in *FD1*-silenced plants. We speculate that the anti-PVX mechanism in FD1-overexpressing transgenic plants may be due to an interference with the function of p25 by FD1. The plan is to investigate this possibility in future work.

## Supplementary data

Supplementary data are available at *JXB* online.

Fig. S1. The localization of FD1 in *N. benthamiana* cells.

Fig. S2. The mRNA level of *FD1* in PVX-infected and p25-containing leaves.

Fig. S3. The change of the physiological state of *FD1-*silenced leaves.

Fig. S4. The index of PVX infection in *FD1-*silenced and control plants.

Fig. S5. The transcription levels of *GLU1*, *Cals1*, and *Cals8* on *FD1-*silenced or control leaves. 

Fig. S6. Other chloroplast-localized genes were silenced by TRV-based VIGS.

Fig. S7. Silencing of four other chloroplast-localized genes influences transcript levels of key genes in the ABA pathway.

Fig. S8. Silencing of four other chloroplast-localized genes influences transcript levels of key genes in the SA-dependent signalling pathway.

Fig. S9. The transcript levels of *GLU1* and *Cals1* in *FD1-*silenced leaves treated with ABA or SA.

Fig. S10. Stable transgenic lines of *N. benthamiana* overexpressing FD1.

Table S1. Primers used in this study.

erz565_suppl_supplementary_table_S1_figures_S1_S10
