# Supplementary material for: Involvement of the chloroplast gene ferredoxin 1 in multiple responses of Nicotiana benthamiana to Potato virus X infection
Source: J Exp Bot. 2019 Dec 24;71(6):2142–56. doi: 10.1093/jxb/erz565 (PMC7094082; doi:10.1093/jxb/erz565)
Supplement: erz565_suppl_supplementary_table_S1_figures_S1_S10 [file erz565_suppl_supplementary_table_s1_figures_s1_s10.pdf]

Supporting information

Supplementary Figure S1

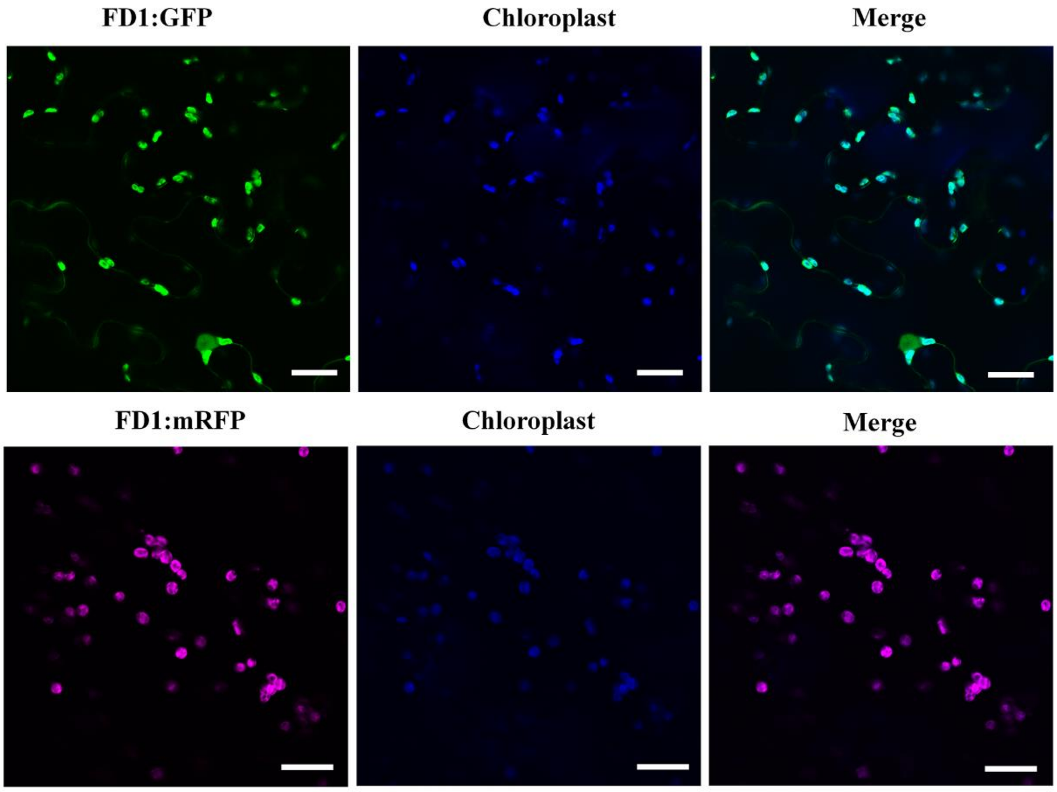

Figure S1. The localization of FD1 in *N. benthamiana* cells. FD1-GFP and FD1-mRFP were agroinfiltrated into *N. benthamiana* leaves, and protein expression was detected by confocal microscopy at 3 dpi. Chloroplast is indicated by blue autofluorescence. Bars = 25 μm.

Supplementary Figure S2

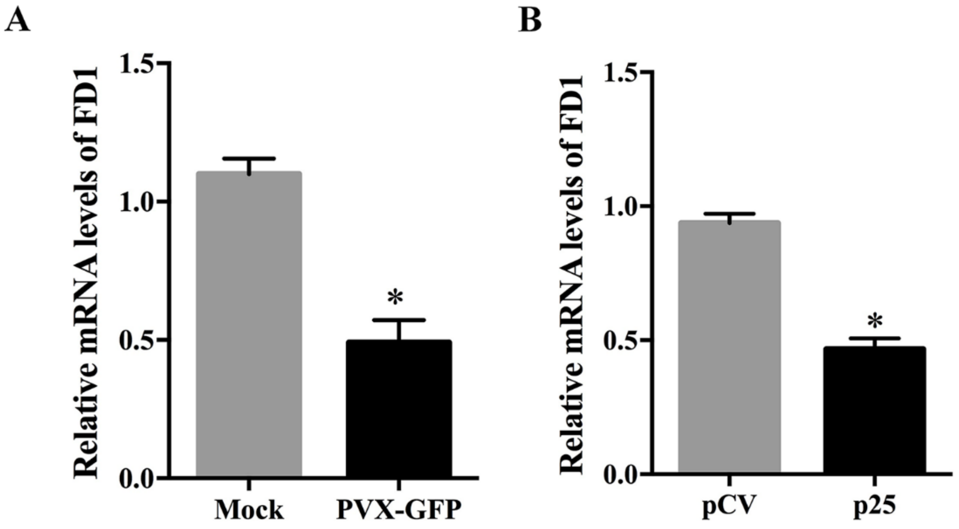

Figure S2. The mRNA level of *FD1* in PVX infected and p25-containing leaves. (A) PVX- infected leaves, (B) leaves infiltrated with expression clones pCV (empty vector) or p25, *FD1* expression measured by qRT-PCR. Results from three repeats are shown. Bars represent the standard errors of the means from three biological repeats. A two-sample unequal variance directional t test was used to test the significance of the difference (*, p value<0.05; **, p value<0.01).

Supplementary Figure S3

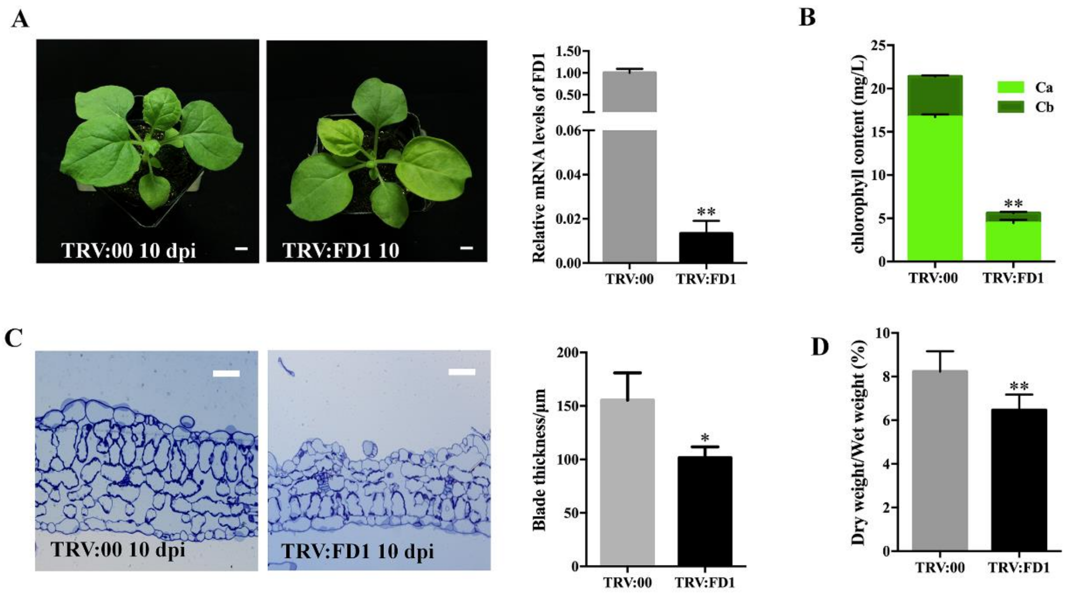

Figure S3. The change of physiological state of *FD1*-silenced leaves.

(A) TRV VIGS was used to silence *FD1* in *N. benthamiana* leaves and photographed at 10 dpi. Bars represents 1 cm. The silencing efficiency of TRV:FD1 was measured by qRT-PCR. (B) The content of chlorophyll in TRV:FD1- and TRV:00-infected leaves was measured using a SpectraMax I3 at 663 nm and 645 nm. C_a = Chlorophyll a; C_b = chlorophyll b (C) longitudinal section of leaves of TRV:00- and TRV:FD1-infected plants stained by toluidine blue. Bar = 20 μm. Leaf blade thickness was measured using ImageJ software. (D) Dry weight and wet weight of TRV:00- and TRV:FD1-infected leaves. Bars represent the standard errors of the means from three biological repeats. A two-sample unequal variance directional t test was used to test the significance of the difference (*, p value<0.05; **, p value<0.01).

Supplementary Figure S4

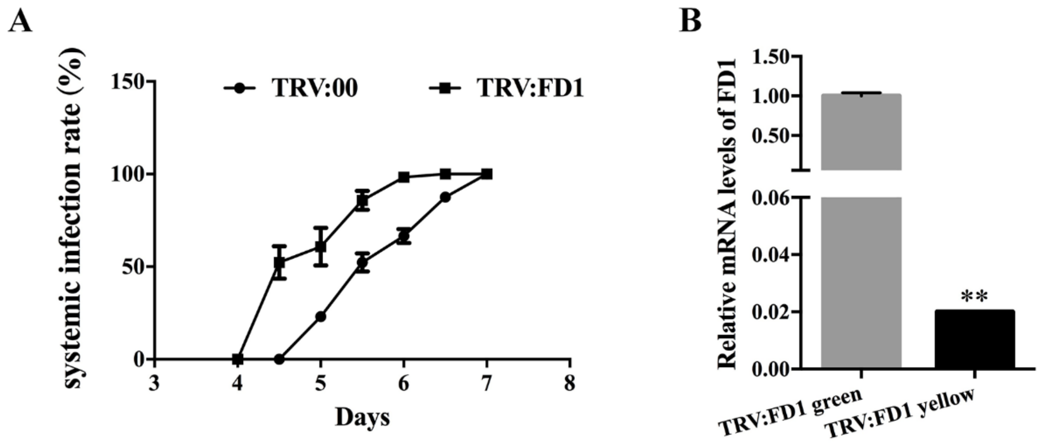

Figure S4. The index of PVX infection in *FD1*-silenced and control plants.

(A) Time-course analysis of PVX-GFP infection in TRV:00- and TRV:FD1-treated plants. Error bars represent the SD for three independent experiments each using 15 plants per treatment. (B) The silencing efficiency of TRV:FD1 in dark green and yellow leaf sectors was measured by qRT-PCR. Bars represent the standard errors of the means from three biological repeats. A two-sample unequal variance directional t test was used to test the significance of the difference (*, p value<0.05; **, p value<0.01).

Supplementary Figure S5

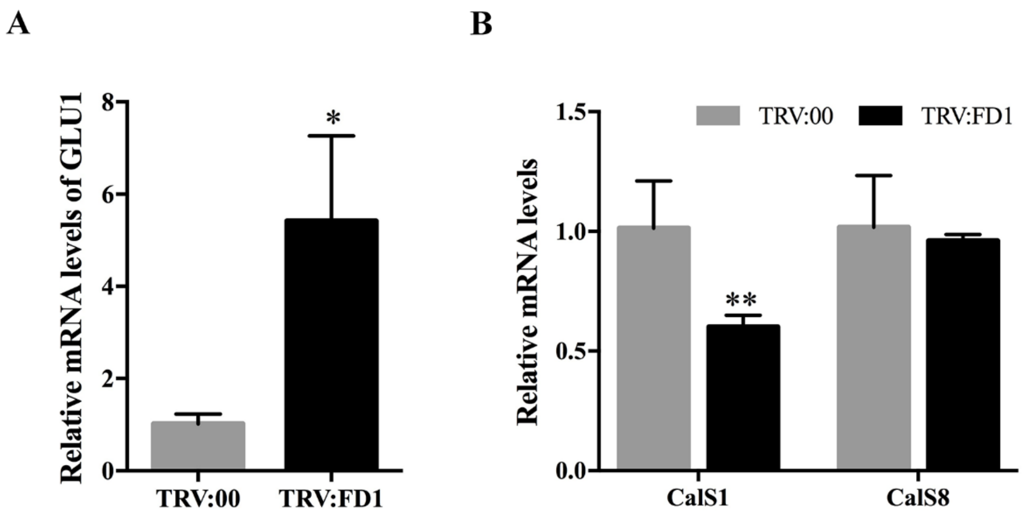

Figure S5. The transcription levels of *GLU1*, *Cals1* and *Cals8* on *FD1* silenced or control leaves.

(A) The transcript levels of (A) *GLU1* and (B) of *Cals1* and *Cals8* in *FD1*-silenced or control leaves was measured by qRT-PCR. Bars represent the standard errors of the means from three biological repeats. A two-sample unequal variance directional t test was used to test the significance of the difference (*, p value<0.05; **, p value<0.01).

Supplementary Figure S6

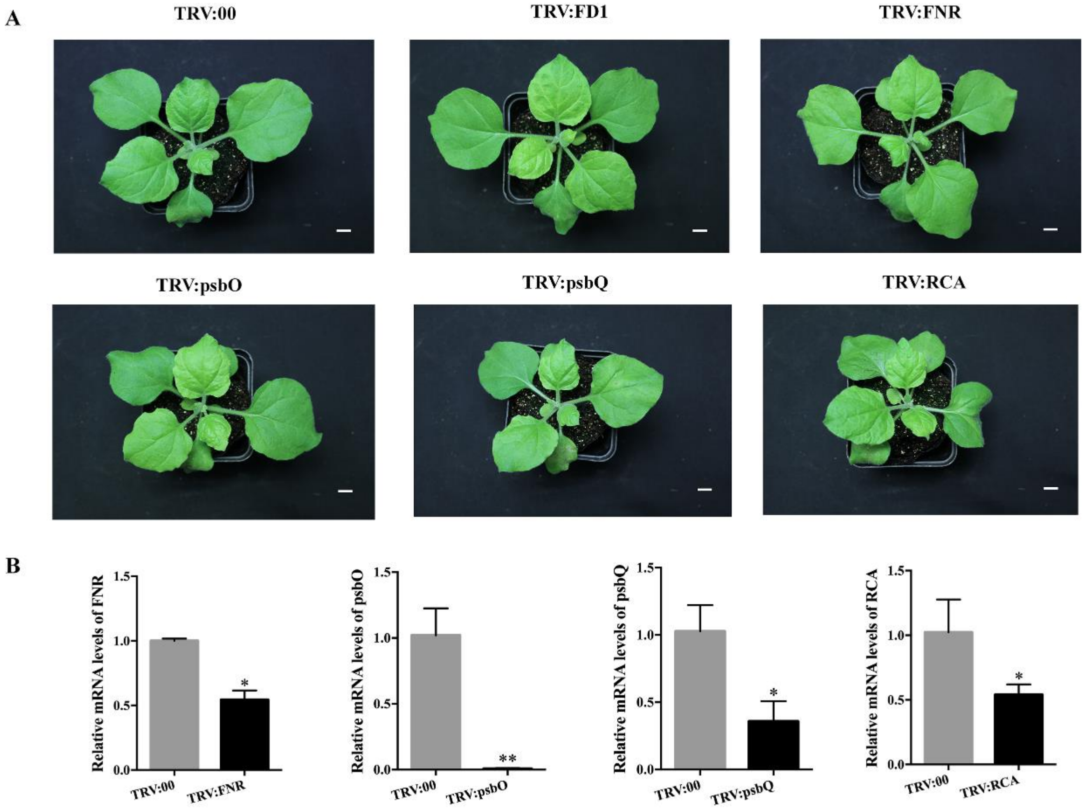

Figure S6. Other chloroplast-localized genes were silenced by TRV based VIGS. (A) TRV VIGS was used to silence *FD1*, *FNR*, *psbO*, *psbQ* and *RCA* in *N. benthamiana* and leaves were photographed at 10 dpi. Bars represents 1 cm. (B) The efficiency of silencing was measured by qRT-PCR. Bars represent the standard errors of the means from three biological repeats. A two-sample unequal variance directional t test was used to test the significance of the difference (*, p value<0.05; **, p value<0.01).

Supplementary Figure S7

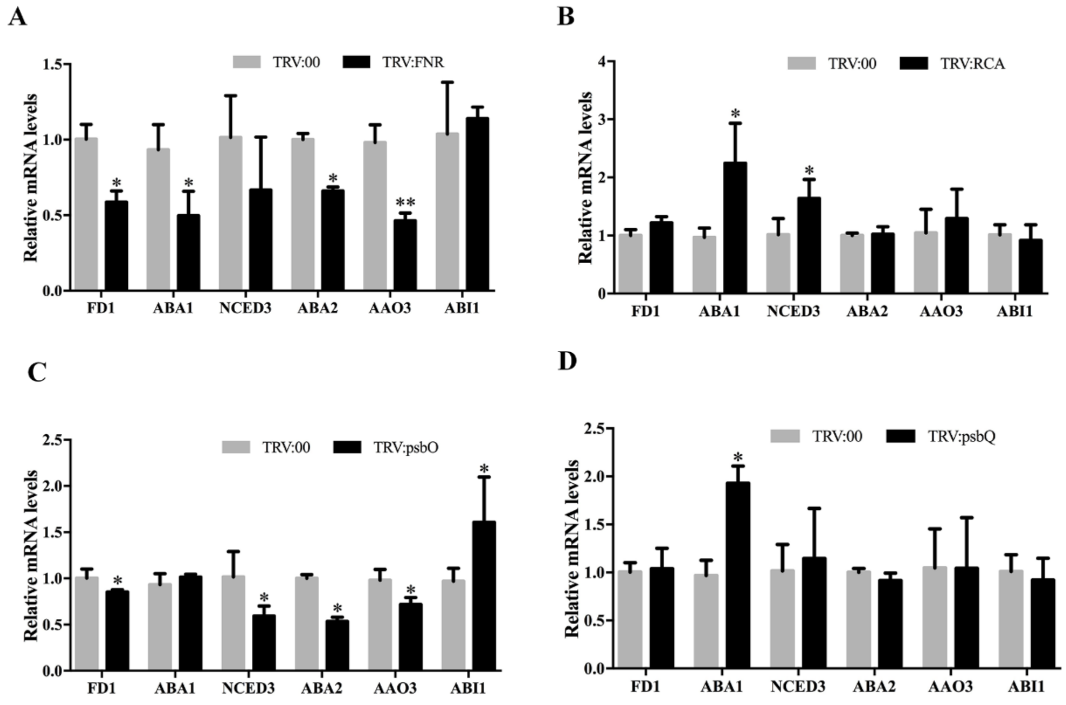

Figure S7. Silencing of four other chloroplast-localized genes influences transcript levels of key genes in the ABA pathway.

(A-D) qRT-PCR was used to measure the transcript levels of the ABA pathway related genes *ABA1*, *NCBD3*, *ABA2*, *AAO* and *ABI1* in TRV:00, TRV:FNR, TRV:psbO, TRV:psbQ and TRV:RCA-treated plants. Bars represent the standard errors of the means from three biological repeats. A two-sample unequal variance directional t test was used to test the significance of the difference (*, p value<0.05; **, p value<0.01).

Supplementary Figure S8

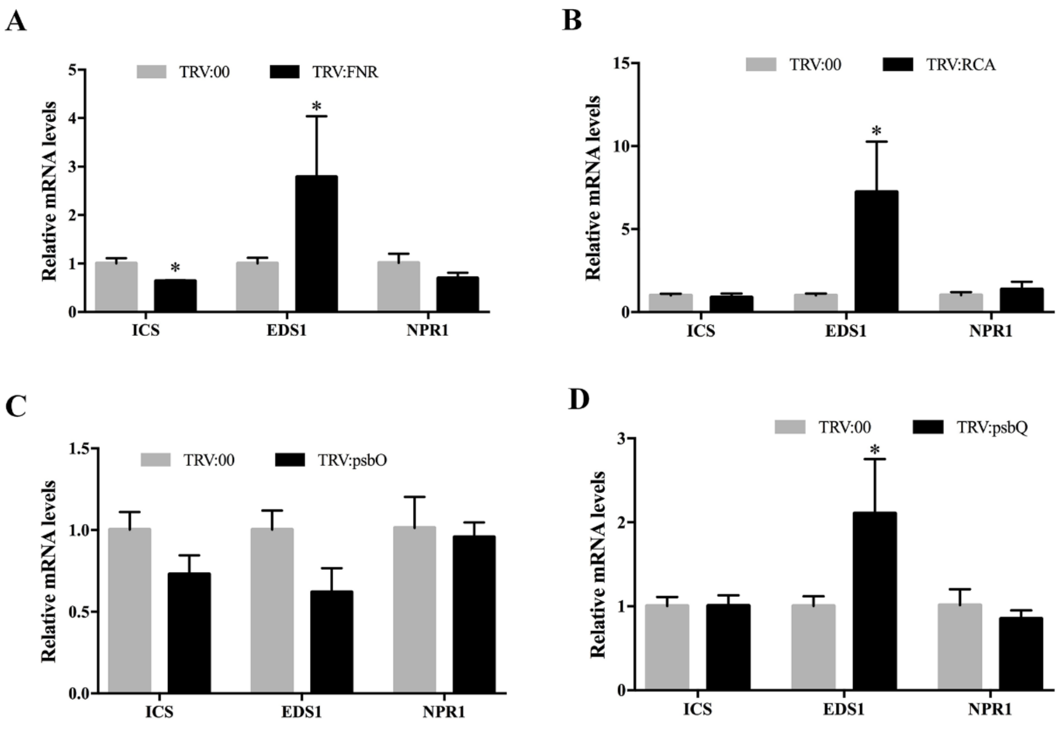

Figure S8. Silencing of four other chloroplast-localized genes influences transcript levels of key genes in the SA-dependent signaling pathway.

(A-D) qRT-PCR was used to measure the transcript levels of the SA-dependent signaling pathway related genes *EDS1*, *ICS1* and *NPR1* in TRV:00, TRV:FNR, TRV:psbO, TRV:psbQ and TRV:RCA-treated plants. Bars represent the standard errors of the means from three biological repeats. A two-sample unequal variance directional t test was used to test the significance of the difference (*, p value<0.05; **, p value<0.01).

Supplementary Figure S9

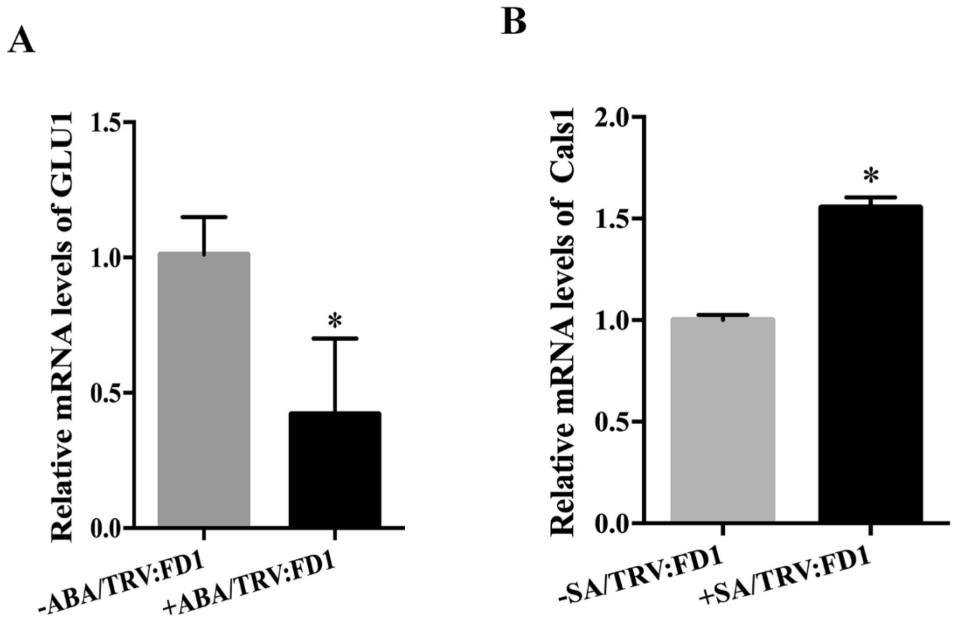

Figure S9. The transcript levels of *GLU1* and *Cals1* in *FD1*-silenced leaves treated with ABA or SA, respectively.

(A) The transcript levels of *GLU1* in *FD1* silenced leaves treated with ABA. (B) The transcript levels of *Cals1* in *FD1* silenced leaves treated with SA. Bars represent the standard errors of the means from three biological repeats. A two-sample unequal variance directional t test was used to test the significance of the difference (*, p value<0.05; **, p value<0.01).

Supplementary Figure S10

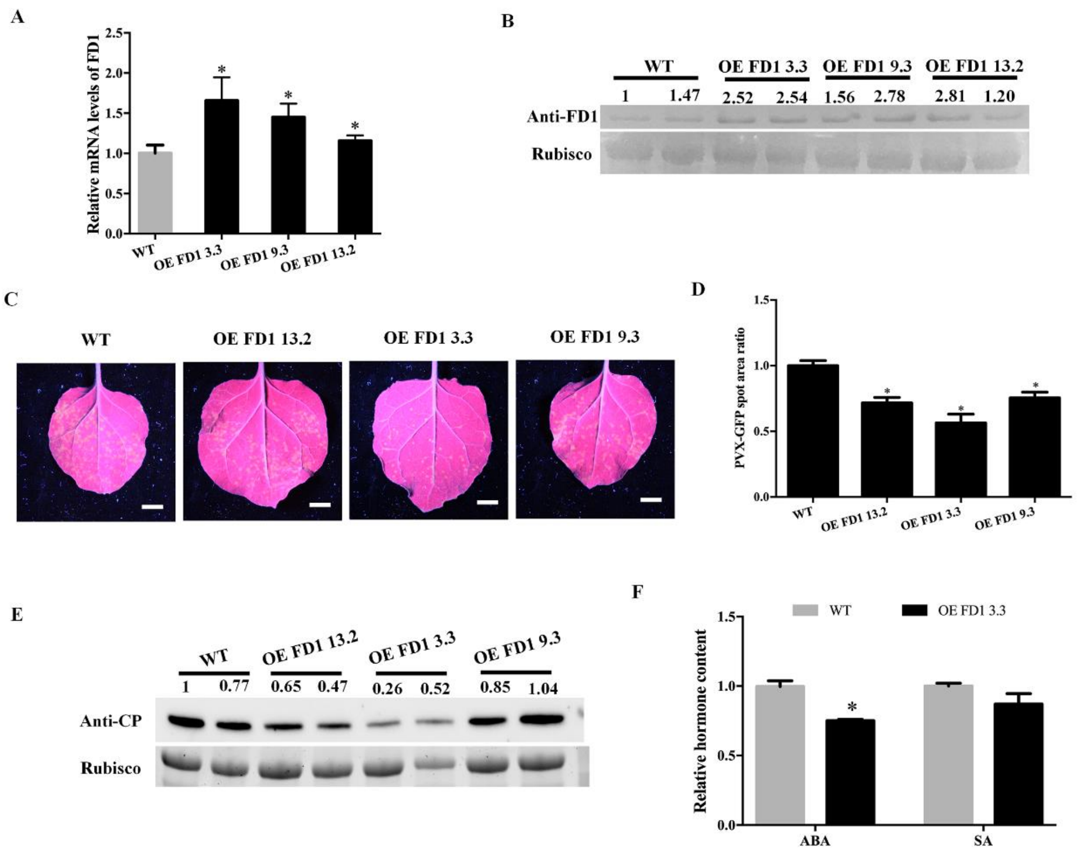

Figure S10. Stable transgenic lines of *N. benthamiana* overexpressing FD1.

(A) The transcript levels of FD1 in three independent transgenic lines OE FD1 3.3, OE FD1 9.3 and OE FD1 13.2. (B) The protein levels of FD1 in three independent transgenic lines were measured by western blotting with a FD1 antibody. (C) Fluorescent foci of PVX-GFP on inoculated leaves of WT and OE FD1 transgenic plants were detected under UV light. Bars represents 1 cm. (D) The area of PVX-GFP infection foci was measured using ImageJ software. (E) PVX CP accumulation in PVX-GFP inoculated leaves of WT, OE FD1 13.2, OE FD1 3.3 and OE FD1 9.3 plants was shown by western blotting with a PVX CP antibody. (F) The relative levels of ABA and SA in OE FD1 3.3 and WT plants was measured by LC-MS. Bars represent the standard errors of the means from three biological repeats. A two-sample unequal variance directional t test was used to test the significance of the difference (*, p value<0.05).

Supplementary Table S1

Table S1. Primers used in this study.

Primers		Use
FD1 VIGS-F	5'- TTGGTCTTAAATCTCAGAGG -3'	VIGS
FD1 VIGS-R	5'- TTAGGCGGTGAGCTCCTC -3'	VIGS
FNR VIGS-F	5'- AATGCCACCGTTATAATGCT -3'	VIGS
FNR VIGS-R	5'- TTAGTAGACTTCAACATTCCA -3'	VIGS
psbO VIGS-F	5'- AATGGAACCCCAGCAAAGAA -3'	VIGS
psbO VIGS-R	5'- ATTTCTTTTTAAGAGGGGGG -3'	VIGS
psbQ VIGS-F	5'- CTTTGAACTCGGATGAGGC -3'	VIGS
psbQ VIGS-R	5'- TTAACCAAGTTTGGCAAGAAC -3'	VIGS
RCA VIGS-F	5'- ATTTCTCCACATTGTATGCTC -3'	VIGS
RCA VIGS-R	5'- CTAACTAGCAAAGAAGGATC -3'	VIGS
UBC RT f	5'- TTTCGGTCCTGATGATACTCCC -3'	qRT-PCR
UBC RT r	5'- CACAGAGCAAAGACTGGATTGA -3'	qRT-PCR
EF1A RT f	5'- GATTGGTGGTATTGGTACTGTC -3'	qRT-PCR
EF1A RT r	5'- AGCTTCGTGGTGCATCTC -3'	qRT-PCR
FD1 RT f	5'- ATGGCCAGTATTTCAGGTAC -3'	qRT-PCR
FD1 RT r	5'- TCAAGAAAGTTTCCATCAGAC -3'	qRT-PCR
FNR RT f	5'- ACTGAGGGAGAGGTCCCA -3'	qRT-PCR
FNR RT r	5'- CTCCAGGCTTCAAGTCACA -3'	qRT-PCR
psbO RT f	5'- CTTGCACCAGCATGCACT -3'	qRT-PCR
psbO RT r	5'- TAGCTGGGACTTGCAACTT -3'	qRT-PCR
psbQ RT f	5'- ACTATGGCTCAAGCTATGG -3'	qRT-PCR
psbQ RT r	5'- TTCCAGGTAATCCACCAGA -3'	qRT-PCR
RCA RT f	5'- AGACCGACAGTGACAGATG -3'	qRT-PCR
RCA RT r	5'- CTTCTCTGTACCTTTGCCTA -3'	qRT-PCR
GLU1 RT f	5'- GGTTGCAAGATCAAATCAAGG -3'	qRT-PCR
GLU1 RT r	5'- TAAGAGTGGAAGGTTATGTCG -3'	qRT-PCR
Cals1 RT f	5'- TTGCATTCTTGCTTTCATGC -3'	qRT-PCR
Cals1 RT r	5'- GGAACTCCGAAACAAATGGA -3'	qRT-PCR
Cals8 RT f	5'- TGAACCACCACTGTTTGCAT -3'	qRT-PCR
Cals8 RT r	5'- CAGAGAAGTGGCGAAAAAGG -3'	qRT-PCR
ABA1 RT f	5'- GTGGAACGTGGACTTCCTGT -3'	qRT-PCR
ABA1 RT r	5'- TACATCACTGGGTCCGAACA -3'	qRT-PCR
NCBD3 RT f	5'- ACCCGACTCGATTTTCAATG -3'	qRT-PCR
NCBD3 RT r	5'- AACTTTTGGCCATGGTTCTG -3'	qRT-PCR
ABA2 RT f	5'- GGTGTTTTCCTGGGAATGAA -3'	qRT-PCR
ABA2 RT r	5'- CAGTTGCAACAGCGTAAGGA -3'	qRT-PCR
AAO3 RT f	5'- CATGAAAGATTTGCCGGTTT -3'	qRT-PCR
AAO3 RT r	5'- ACTTGCAAGCATCAGCAATG -3'	qRT-PCR

ABI1 RT f	5'- GCCGTTGTTTGTTCATCTC -3'	qRT-PCR
ABI1 RT r	5'- ACTGTATCACCTTGCCTCC -3'	qRT-PCR
EDS1 RT f	5'- AGGCCGAAGCGTTATAGGTT -3'	qRT-PCR
EDS1 RT r	5'- AAAACATCATCGCCCAGAAG -3'	qRT-PCR
ICS1 RT f	5'- AGACTCCGGACTGAAGACGA -3'	qRT-PCR
ICS1 RT r	5'- CCAAGACCCTTTTCAACCAA -3'	qRT-PCR
NPR1 RT f	5'- TAGCGTTCCTTGCTGAGGTT -3'	qRT-PCR
NPR1 RT r	5'- CTCTCGCATGCTTTACCACA -3'	qRT-PCR
PR1 RT f	5'- TGAGATGTGGGTCGATGAGA -3'	qRT-PCR
PR1 RT r	5'- GGACGCCTTTTCAATTCGTA -3'	qRT-PCR
probe-PVX CP f	5'- TGGGACTTAGTCAGACACT -3'	probe
probe-PVX CP r	5'- ACCTCGAGTGACAGCTGC -3'	probe
probe-FD1 f	5'- TTAGAGGGGTGGTAGGATTACTTGC -3'	probe
probe-FD1 r	5'- TGGGTAAGCAACACAGGTTAGCAC -3'	probe
